# Effect of megarectum on postoperative defecation of female patients with congenital rectovestibular fistula or rectoperineal fistula

**DOI:** 10.3389/fped.2023.1095054

**Published:** 2023-03-27

**Authors:** Chunxiang Liu, Song Wang, Jinyu Dai, Jian Li, Xiaoxia Wu, Yong Liu, Zhiwei Yao, Lushun Ma, Xiaobing Sun, Daqing Sun

**Affiliations:** ^1^ Department of Pediatric Surgery, Tianjin Medical University General Hospital, Tianjin, China; ^2^ Department of Pediatric Surgery, Shanxi Children’s Hospital, Taiyuan, China; ^3^ Department of Pediatric Surgery, Shanxi Bethune Hospital, Taiyuan, China

**Keywords:** anorectal malformation, rectovestibular fistula, rectoperineal fistula, megarectum, defecation

## Abstract

**Background:**

To assess the effect of megarectum on postoperative defecation of female patients with congenital rectovestibular fistula or rectoperineal fistula.

**Methods:**

From March 2013 to February 2021, 74 female patients with congenital rectovestibular fistula or rectoperineal fistula were treated. The age of patients ranged from 3 months to 1 year. Barium enema and spinal cord MRI were performed in all children. 4 patients were removed from the study because of spinal cord and sacral agenesis. Finally, 70 patients underwent one-stage anterior sagittal anorectoplasty (ASARP). Anal endoscopy and anorectal pressure measurement were performed 1 year after surgery. All patients were divided into two groups depending on the presence of megarectum (+) and (−) and observed for constipation and anal sphincter function.

**Results:**

16 patients (4 months to 1 year) were complicated with megarectum, and 5 patients (3 months to 9 months) were without megarectum. The incision infection was seen in 3 patients. All patients were followed up for 1 year to 5 years. Fecal soiling was seen in 2 patients and constipation in 14 patients. Among 16 patients with megarectum, soiling was seen in 1 patient and the constipation in 12 patients. Among 54 patients without megarectum, soiling was seen in 1 patient and constipation in 2 patients. There was a significant difference in the incidence of postoperative constipation between the two groups (megarectum (+) 75% vs. megarectum (−) 3.7% (*P* < 0.05)). However, there was no significant difference in the score of anal sphincters between the two groups (*P* < 0.05). And there was no significant difference in anal resting pressure (*P* = 0.49) and length of anal high pressure area (*P* = 0.76). 7 patients with constipation and megarectum acquired normal anal function after the dilated rectum was resected.

**Conclusion:**

Megarectum increases the possibility of difficult postoperative defecation in the patients with congenital rectovestibular fistula or rectoperineal fistula. However, constipation was not associated with ASARP postoperative effects on sphincter function. Resection of megarectum is helpful to the improvement of constipation.

## 1. Introduction

Congenital rectovestibular fistula and rectoperineal fistula are the most common anorectal malformations (ARM) in female newborns. The operative methods to correct these malformations include posterior sagittal anorectoplasty (PSARP) (1), anterior sagittal anorectoplasty (ASARP) (2–4), and neutral sagittal approach anorectoplasty (NSARP) (5). However, Nam et al. reported that the incidence of constipation was as high as 30.7% after anorectoplasty (6). Consistent with this, Chang et al. found sixty-five out of 84 (77.4%) patients with constipation after ARM repair and eighteen of 65 patients with megarectosigmoid (7). Although the incidence of constipation after ARM surgery is very high, and whether it is related to megarectosigmoid still needs to be explored. In addition, the satisfactory effect of surgical resection of megarectosigmoid on constipation, is still controversial. Some studies suggest that aggressive medical treatment is comparable with the treatment of resecting the megarectosigmoid (7, 8).

Therefore, we followed up 70 children with congenital rectovestibular fistula or rectoperineal fistula who underwent one-stage ASAPR. The aim of this study is to investigate the effect of preoperative megarectum on postoperative defecation and whether the megarectum necessitated surgical resection.

## 2. Materials and methods

### 2.1. Data sources and patient selection

**Figure 1:**
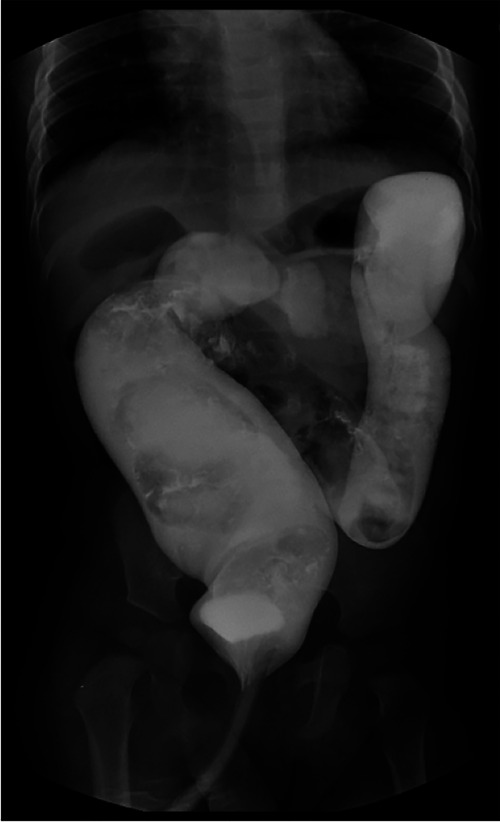
Barium enema indicates megarectum.

The data of children with ARM admitted to Tianjin Medical University General Hospital, Shanxi Children's Hospital and Bethune Hospital of Shanxi Province from March 2013 to February 2021 were collected and followed up. The study has been approved by the ethics committee of Tianjin Medical University General Hospital, Shanxi Children's Hospital and Bethune Hospital of Shanxi Province. Children aged between 3 months and 1 year old were included in the study. All patients underwent barium enema and spinal cord MRI examination, and then were performed one-stage ASAPR. After excluding 4 cases of spinal cord dysplasia or sacral hypoplasia, a total of 70 children were included in this study. Barium enema showed that 16 cases were complicated with megarectum (rectopelvic ratio >0.61, Figure 1).

### 2.2. Evaluation method

All the children were given clean enema before surgery and stopped feeding for one day to full preparation of the intestinal tract. Preoperatively, rectal washouts were administered. Preoperative antibiotic prophylaxis was given in the operating room and was continued for prophylactic treatment (24 h). Anal dilatation was started 2 weeks after the operation. Anal endoscopy and anorectal pressure measurement were performed 1 year after surgery. Anal endosonography was performed by HITACHI EUB7000 (360° scan probe, diameter 12 mm, frequency 5–10 Hz). The sphincter integrity and the degree of the injury were evaluated by endoanal ultrasound, using the sphincter defect thickness and defect extent score: 1 was classified as mild injury, 2–3 as moderate injury, and 4–5 as severe injury, as shown in Table 1. Anorectal manometry was evaluated by High Resolution Manometry system (ManoScanTM, Serial Technique Instruments, United States).

**Table 1:** Scoring standard of sphincter damage degree.

Parameter	Grade (score)					
	0	1	2	3	4	5
Defect thickness	No defect	Partial defect	Total defect			
Defect range	0	≤45°	45–90°	90–135°	135–180°	>180°

### 2.3. Statistical analysis

The incidence of constipation and sphincter damage were compared between the patients with megarectum and the patients without megarectum by *χ*^2^ test. Anorectal manometry was compared between the two groups by *t*-test. SPSS Statistics 22. (IBM, Armonk, NY) was used for statistical analysis. Data are expressed as average ± SDs, number (percentage). The *P*-value <0.05 was considered statistically significant.

## 3. Results

Sixteen (22.9%) out of 70 patients were associated with congenital giant rectum and 54 (77.1%) were not associated with congenital giant rectum. All patients were followed up for 1–5 years after ASARP. Among 16 patients with megarectum, soiling was seen in 1 patient and the constipation in 12 patients. Among 54 patients without megarectum, soiling was seen in 1 patient and constipation in 2 patients. As shown in Table 2, there was a significant difference in the incidence of postoperative constipation between the two groups (megarectum (+) 75% vs. megarectum (−) 3.7% (*P* = 0.00)). The incidence of constipation in the group with giant rectum was significantly higher than that in the group without giant rectum. This suggests that preoperative megarectum is an important cause of postoperative constipation. According to the score of anal sphincters, the sphincter was mildly damaged in 7 patients and intact in 9 patients in the megacolon group, and mildly damaged in 16 patients and intact in 38 patients in the non-megacolon group. As shown in Table 3, there was no significant difference in the score of anal sphincter damage between the two groups (*P* = 0.29). In addition, as shown in Table 4, there was no significant difference in anal resting pressure (*P* = 0.49) and length of anal high pressure area (*P* = 0.76). This suggests that constipation and soiling are not related to sphincter function. Of the 16 patients with constipation, 7 underwent two-stage giant rectal resection and all returned to normal defecation.

**Table 2:** Comparison of postoperative defecation function between the two groups.

Group	Number of cases	Constipation	No constipation
Megarectum	16	12	4
No megarectum	54	2	52

Continuous correction *χ*^2^ test *χ*^2^ = 34.9, *P < *0.05.

**Table 3:** Comparison of postoperative sphincter score between the two groups.

Group	Number of cases	Complete	Mild damage
Megarectum	16	9	7
No megarectum	54	38	16

Continuous correction *χ*^2^ test *χ*^2^ = 1.12, *P* = 0.29.

**Table 4:** Comparison of anorectal manometry between the two groups after operation.

Group	Cases	Anal resting pressure (mmHg)	Length of high pressure area of anal canal (cm)
Megarectum	16	31.74 ± 3.11	1.20 ± 0.13
No megarectum	54	30.96 ± 3.33	1.21 ± 0.09
*t*		0.69	0.30
*p*		0.49	0.76

## 4. Discussion

PSARP is a surgical method for the treatment of ARM proposed by de Vries and Peña in 1982 (1). The advantage of this procedure is to provide complete exposure of the anorectal region by means of a median sagittal incision from sacrum to fistula and muscle structures of pelvic floor can be reconstructed under direct vision. In addition, the incision is in the middle of the pelvic floor which can prevent the damage to the pelvic floor the nerve and blood vessels on the pelvic floor. In 1992, Okada et al. (4) proposed ASARP in which only anterior sphincter complex was incised. ASARP makes smaller incision compared to PSARP and the dissection of the rectum and vagina is clearer (2). In addition, incisions are easier to manage after ASARP. Therefore, ASARP has become one of the main surgical methods for the treatment of ARM and has been widely accepted. In addition, Dave and Shi (5) modified the operation of ASARP and presented an anal transposition procedure which is described as NSARP. NSARP preserves a perineal a skin bridge between the anus and the fistula. The main advantage is that this procedure does not damage the skin and tissue between the anus and the vagina.

To avoid wound infection and subsequent anal retraction, anal stenosis or recurrence of fistula, De Vries and Peña initially advocated three-staged operations: colostomy primarily, posterior sagittal anorectoplasty secondly, and closure of colostomy thirdly. In recent years, more pediatric surgeons prefer one stage surgery in the neonatal period. Although the risk of wound infection may be higher, it can reduce the numbers of operation, the cost of medical treatment and it can avoid the complications resulting from colostomy, such as skin erosion, wound infection, prolapse or stenosis of stoma and disturbance of water and electrolyte homeostasis (9–11). In fact, if adequate intestinal preparation and prophylactic use of antibiotics are made before operation, one-stage surgery is safe with fewer complications (10, 11). As for the timing of the operation, many doctors think that it can be completed in the neonatal period (9, 11). Some doctors think that the anal reconstruction during the neonatal period contributes to the early establishment of brain-defecation reflex, pelvic floor muscle training, synapse and neural network formation and results in normal or near-normal anal function (12). In our study, all patients underwent one stage ASAPR after 3 months old.

Previous literature suggests that all kinds of operative approaches produce satisfactory results no matter whether it is one-stage or multiple-stage and whether it is in the neonatal period or later. However, constipation often develops after operation (3, 8, 13). Kulshreshtha et al. (3) reported that 63 cases (58.8%) out of 107 cases had constipation 3 months later. Zhang et al. (11) reported that the incidence of constipation after ASARP was 57.7%. Defecation control involves coordination among several different neural pathways, pelvic floor muscles and rectum motility. In addition, fecal volume and viscosity, colonic transport capacity, rectal compliance, rectal peristalsis, anorectal angle, anorectal sensation, reflex mechanism, pelvic floor muscle integrity and other factors also affect defecation. The causes of postoperative constipation include anal stenosis, megarectum (12, 13) and levator ani dysplasia (14), pelvic floor dissynergia (15), and spinal cord dysplasia (16).

**Figure 2:**
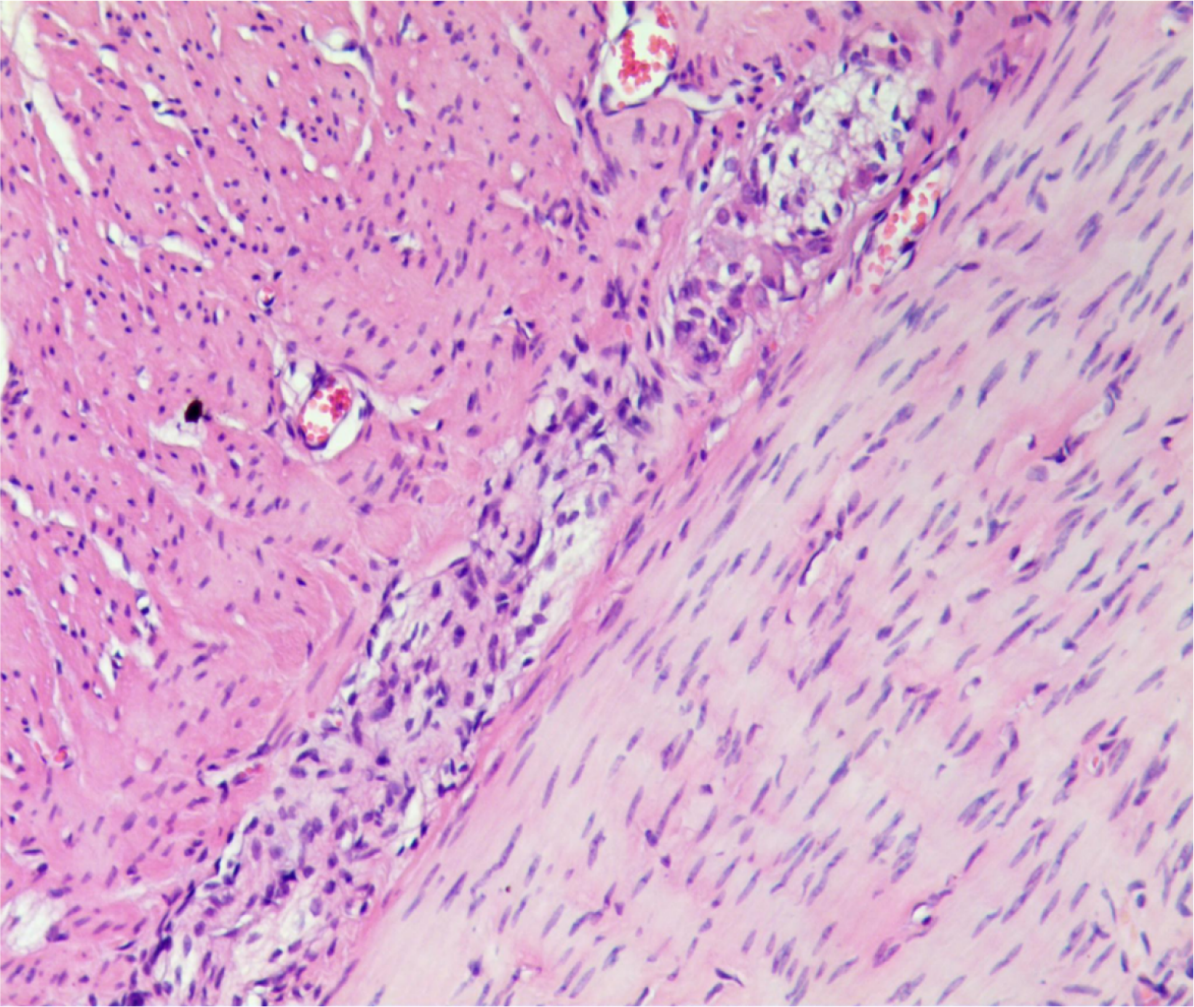
Proliferation of nerve fibers in the myenteric plexus of dilated intestine. A small number of less developed ganglion cells can be seen. ( ×40).

Some studies suggest that giant rectum is the cause of intractable constipation after anorectopalasty (8, 14). Megarectum is an enlarged rectum defined by a rectopelvic ratio greater than 0.61 and with significant abnormalities in anorectal manometry, pressure-volume curves, or rectal compliance investigation (14). In children with megarectum, rectal volume increases and rectal sensory and peristaltic function decreases, resulting in fecal retention in the rectum and constipation. In our study, 16 out of 70 patients had megarectum. There were no significant differences in postoperative sphincter score, anal resting pressure and the length of anal high pressure area between these 16 patients and other 54 patients without megarectum. However, 12 of 16 patients with megarectum developed intractable constipation after operation, while only 2 of other 54 patients developed postoperative constipation. Our clinical results indicated that megarectum might be an important cause of postoperative constipation in patients with congenital rectovestibular fistula or perineal fistula. Bhatia et al. (17) reported that the rectal Cajal interstitial cells and ganglion cells were reduced in children with AMR, and the content of calretinin was also reduced (Figure 2). Mandhan et al. (18) found that the decreased expression of neuron-specific enolase, vasoactive intestinal peptide and substance *P* in the rectum of AMR fetal rats may lead to rectal motility disturbance and become the pathological basis of constipation. Li et al. (19) discovered the pathological changes including decreased ganglion cells, degeneration of myocyte hyaline and moderate fibrosis in the megarectum and suggested that excision of megarectum could be effective in the treatment of constipation. In this study, among the above-mentioned 12 children with postoperative constipation, 7 patients underwent secondary excision of megarectum, and then the constipation disappeared. These results suggested that excision of megarectum should be performed at the same time with anorectoplasty to avoid defecation in children with rectovestibular fistula or rectoperineal fistula.

The mechanism behind the development of megarectum is not clear in the patients with rectovestibular fistula or rectoperineal fistula. In this study, the children were treated late, and the megarectum may be secondary to defecation difficulties. De la Torre et al. (20) have reported that megarectum can also occur in neonatal period. We think that the anorectoplasty can be completed in the neonatal period primarily for the children who are diagnosed with rectovestibular fistula or rectoperineal fistula after birth. If the operation is performed after neonatal period, the enema treatment should be performed daily before the surgery, as well as dilatation of the fistula to avoid the secondary megarectum. In addition, preoperative barium enema should be performed for the children with rectovestibular fistula or rectoperineal fistula to exam the existence of megarectum. It is very likely that the children with megarectum may have difficult postoperative defecation. Therefore, in the case of combined megarectum, it is recommended to remove the megarectum at the same time during the operation to avoid postoperative constipation and reoperation.

## 5. Conclusion

Megarectum increases the possibility of difficult postoperative defecation in the patients with congenital rectovestibular fistula or rectoperineal fistula. However, constipation was not associated with ASARP postoperative effects on sphincter function. Resection of megarectum is helpful to the improvement of constipation.

## Data Availability

The original contributions presented in the study are included in the article/Supplementary Material, further inquiries can be directed to the corresponding author/s.
